# A meta-analysis of weekly cisplatin versus three weekly cisplatin chemotherapy plus concurrent radiotherapy (CRT) for advanced head and neck cancer (HNC)

**DOI:** 10.18632/oncotarget.11824

**Published:** 2016-09-02

**Authors:** Jian Guan, Yue Zhang, Qinyang Li, Yaowei Zhang, Lu Li, Min Chen, Nanjie Xiao, Longhua Chen

**Affiliations:** ^1^ Department of Radiation Oncology, Nanfang Hospital, Southern Medical University, Guangzhou, China

**Keywords:** cisplatin, head and neck, cancer, therapy, meta-analysis

## Abstract

**Purpose:**

This study was performed to compare the efficacies and acute toxicities in weekly- and three weekly- cisplatin based concurrent chemoradiotherapy (CCRT) for advanced HNC patients.

**Results:**

779 patients of 10 studies were eligible. No difference in the 2-, 3-year OS or 1-, 2-year LRFS was observed, whereas patients in three weekly CCRT arm tended to have a better 5-year OS (HR=1.79, 95%C 0.97-3.31, p=0.06). Weekly arm seemed to show less gastrointestinal toxicities (RR=0.59, 95%CI 0.34-1.02, p=0.06), but similar hematologic toxicity compared to three weekly arm. Subgroup analysis displayed more grade ≥3 mucositis (RR=1.72, p=0.01), and more chemotherapy related delay/interrupt (RR=2.68, p<0.0001) in weekly arm for non-nasopharynx carcinoma (non-NPC) HNC.

**Methods:**

We conducted the meta-analysis by searching PubMed, MEDLINE, ScienceDirect, Cochrane Library and China National Knowledge Infrastructure (CNKI) databases. The primary endpoint was overall survival (OS) with secondary endpoints locoregional recurrence-free survival (LRFS) and grade≥3 acute adverse events. RevMan 5.2 was used to perform statistical analyses.

**Conclusions:**

Three weekly cisplatin-based CCRT might achieve a higher long-term OS with no significant difference in a shorter OS and LRFS. Weekly arm was associated with less gastrointestinal toxicities but more grade≥3 mucositis and chemotherapy related delay/interrupt. Large randomized trials were urgent to further define superiority of these two regimens.

## INTRODUCTION

Concurrent platinum-based chemoradiation is currently the most widely used regiment for advanced HNC, which provides a significant improvement in 5-year OS compared with radiotherapy alone [1–3]. A 100 mg/m^2^ dose of cisplatin administered once every 3 weeks is the preferred therapeutic regimen as category 1 in NCCN Guidline of head and neck cancers (Version 1.2015), achieving 71% complete response (CR) rate and 34% 4-year survival [4]. But its high emetic potential, hepatotoxicity and nephrotoxicity demand further efforts be made towards improving its therapeutic and toxicity profiles. Various alternative dosing schedules were adopted to deliver cisplatin with concurrent radiotherapy to improve compliance and the toxicity profile. Among these regimens, weekly cisplatin doses ranging from 30 to 40 mg/m^2^ were used most widely, with a CR rate of 80.5% and 3-year OS of 62% [5].

Multiple studies compared the outcomes of the weekly and three-weekly cisplatin-based CCRT in advanced HNC. A randomized control trial was conducted for patients with advanced oral squamous cell carcinoma, showing higher compliance and lower acute toxicity in three-weekly arm [6]. Though another retrospective study showed statistically similar response rates and toxicities between the two arms in patients with stage III/IV head and neck squamous cell carcinoma (HNSCC) [7]. Several other studies also compared the efficacies and toxicities in the two regimens, but none of those were sufficiently to demonstrate the priority of the two schedules in cisplatin as part of CCRT for SCCHN. Therefore, we performed this meta-analysis to provide an assessment on survivals and adverse effects between the different cisplatin schedules for HNC patients.

## RESULTS

### Description of studies

**Figure 1:**
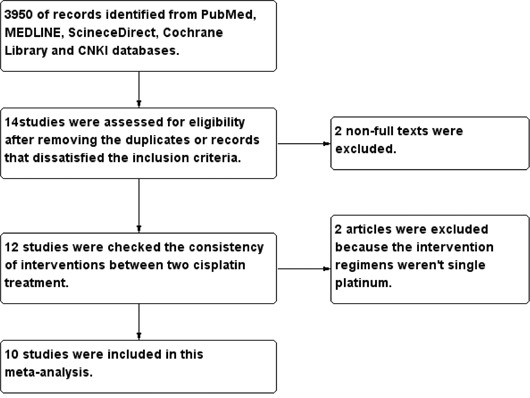
Study selection flow about the cisplatin-based chemoradiotherapy of head and neck cancer CNKI = China National Knowledge Infrastructure.

The preliminary literature screening yielded 3950 records from the five databases. Finally 10 studies [6–15] of 779 patients (376 in weekly cisplatin group and 403 in three weekly group, respectively) were eligible for the meta-analysis published from 2006 to 2014. Almost all patients were stage II-IVb disease with head and neck cancer. Seven studies of enrolled were mainly mentioned of non-nasopharyngeal carcinoma (non-NPC) [6–11, 14], and the other three of all patients were diagnosed with nasopharyngeal carcinoma (NPC) [12–13, 15]. The retrieval flow was performed in the Figure 1. The main characteristics of the included studies were listed in the Table 1.

**Table 1:** Summary of the included studies

Author, year	Method	Country	Stage	N(Q1,Q3)	Age(Q1,Q3, y)	Sex(Q1:M/F; Q3:M/F)	Chemotherapy	Mean total radiation dose
**Geeta SN, 2006**	Retro 2004-05	India	II-IV	32 51	57.5 55	Q1:26/6 Q3:37/14	Q1:40mg/m^2^,6cycles Q3:100mg/m^2^,2-3days,3cycles	66-70Gy 33-35F
**Ho KF, 2008**	Retro 2000-04	England	IVa	24 27			Q1:33-40mg/m^2^,6cycles Q3:80-100mg/m^2^,3cycles	60-70Gy 33F, 45 days
**Huang DN, 2009**	RCT 2003-07	China	III~IVa	33 32	43 41	Q1:24/9 Q3:20/12	QW:30mg/m^2^,7-8cycles Q3W:80mg/m^2^,3cycles	50-76Gy 2Gy/F, 5 days/week
**Uygun K, 2009**	Retro 2002-07	Turkey	III-IV	20 30	71 53.2		Q1:40mg/m^2^,6cycles Q3:100mg/m^2^,3cycles	66-70Gy 33-35F,2Gy/day
**Kose F, 2011**	Retro 2007-09	Turkey	II-IV	32 23	58 60	Q1: 26/6; Q3:18/5	Q1:30mg/m^2^ Q3:100mg/m^2^	50-70Gy 2Gy/day, 5days/week
**Tsan DL, 2012**	RCT 2008-10	Taiwan	II-IV	24 26	49 49.2	Q1:23/1 Q3:25/1	Q1:40mg/m^2^ Q3:100mg/m^2^	66Gy 2Gy/F, 5 days/week
**Espeli V, 2012**	Retro 2002-09	Switzerland	I-IV	40 54	65 58	Q1:32/8 Q3:43/11	Q1:40mg/m^2^,6cycles Q3:100mg/m^2^, 3cycles	66-72Gy
**Jagdis A, 2014**	Retro 2000-09	British	II-IVb	45 28	51 49.5	Q1:35/10 Q3:15/13	Q1:40mg/m^2^ Q3:100mg/m^2^,3cycles	66-70Gy 33-35F
**Geiger JL, 2014**	Retro 2004-10	United States	III-IV	53 51	61 53		Q1:25-30mg/m^2^ Q3:100mg/m^2^,3cycles	60-70Gy 30-35F
**Tao CJ, 2014**	Retro 2003-07	China	II-IVb	73 81	P=0.351	Q1:56/17 Q3:59/22	Q1:30-40mg/m^2^,5-7cycles Q3:80mg/m^2^,3cycles	60-68Gy 30F, 2.27Gy/F

RCT = randomised controlled trial; Retro = retrospective comparative study; Q1=weekly; Q3=three weekly; y=year; M=male; F=female; F=fraction.

### Effects of interventions

#### Survival events

##### Overall survival

**Figure 2:**
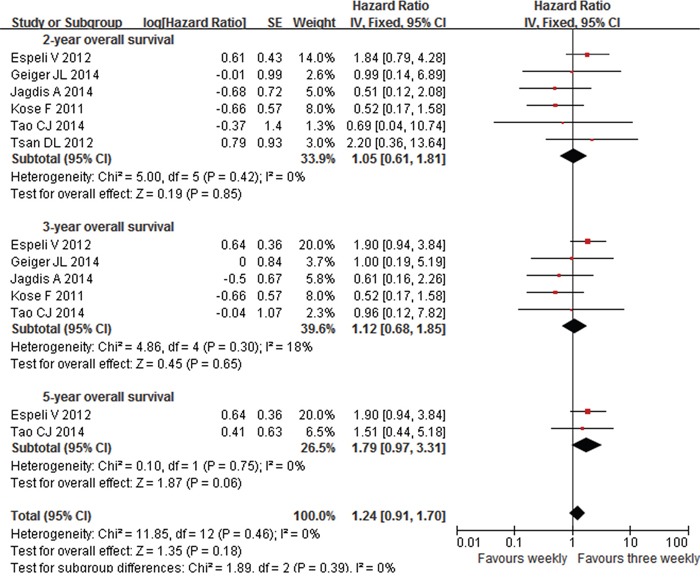
Forest plots of hazard ratios for 2-year, 3-year and 5-year OS in patients between weekly and three weekly cisplatin chemoradiotherapy OS=overall survival. CI=confidence interval, I2=index of heterogeneity.

Six studies [6–7, 9, 13–15] reported the data of OS, which included 267 patients in the weekly group and 263 patients in the three weekly group. Not all of the year-OS data were reported or extractable from all of the included studies. Similar outcome of 2-year OS was observed in the two arms with a HR of 1.05 including 530 eligible patients (95%CI 0.61-1.81, p=0.85), as well as 3-year OS with a HR of 1.12 including 480 eligible patients (95%CI 0.68-1.85; P=0.65) (Figure 2). Patients in three weekly regimens tended to have a better 5-year OS despite the difference had not yet reached the statistical level (HR=1.79, 95%C 0.97-3.31, p=0.06) (Figure 2). Further subgroup analysis displayed no significant difference between the two interventions either for NPC (2-OS: HR=0.54, 95%CI 0.15-1.90, p=0.34; 3-OS: HR=0.69, 95%CI 0.23-2.10, p=0.51) [13, 15] or non-NPC patients (2-OS: HR=1.23, 95%CI 0.67-2.23, p=0.51; 3-OS: HR=1.27, 95%CI 0.72-2.22, p=0.41) [6–7, 9, 14] in terms of 2- and 3-year OS. There was no heterogeneity between studies for the OS analyses.

#### Locoregional recurrence-free survival

**Figure 3:**
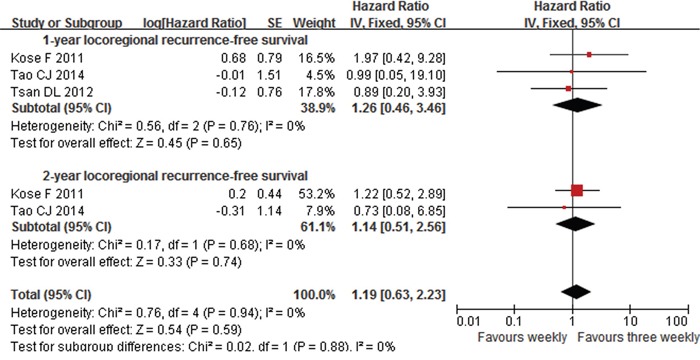
Forest plots of hazard ratios for 1-year and 2-year LRFS in patients between weekly and three weekly cisplatin chemoradiotherapy LRFS=Locoregional recurrence-free survival. CI=confidence interval, I^2^=index of heterogeneity.

Three studies [6, 14–15] were included in the LRFS analysis, including 129 patients in weekly group and 130 patients in three weekly group. Forest plot showed no difference of 1- and 2-year LRFS between the weekly and three weekly cisplatin-based CCRT for HNC patients (1-year LRRFS: HR=1.26, 95%CI 0.46-3.46, p=0.65; 2-year LRRFS: HR=1.14, 95%CI 0.51-2.56, p=0.74). Details were displayed in the Figure 3.

#### Adverse effects

Grade≥3 adverse effects were gathered from the enrolled studies, including hematologic toxicity (neutropenia, thrombocytopenia and anemia) and non-hematologic toxicity, including gastrointestinal toxicity, dermatitis, mucositis.

#### Grade≥3 hematologic toxicity

Six studies supplied data of grade≥3 neutropenia [6, 8, 10–13] among which included 178 patients in the weekly group and 194 patients in the three weekly group. Forest plot showed that patients in the two arms had similar risk of neutropenia and thrombocytopenia with RR of o.85 and 1.13, respectively (95%CI 0.49-1.48, p=0.57; 95%CI 0.42-3.01, p=0.81) (Supplementary Figure S1 and S2). But a trend of anemia risk reduction was observed in favor of the weekly arm with an RR of 2.88(95%CI 0.84-9.94, p=0.09) (Supplementary Figure S3).

#### Grade≥3 gastrointestinal reactions

Nausea and/or vomiting were the most common gastrointestinal reactions for patients treated with ciplatin-based CRT. The data of grade≥3 nausea/vomiting were extracted from six eligible studies [6, 10–13, 15] including 219 patients in weekly group and 224 patients in three weekly group. Patients treated with three weekly cisplatin seemed to be more prone to occur nausea and/or vomiting than those with weekly (RR=0.59, 95%CI 0.34-1.02, p=0.06) (Supplementary Figure S4).

#### Grade≥3 dermatitis

Six eligible studies [6, 8–11, 15] had the data for grade≥3 dermatitis, which included 213 patients in weekly group and 269 patients in three weekly group. The weekly arm appeared similar risk of dermatitis compared to three weekly arm, with an RR of 1.23 (95%CI 0.84-1.82, p=0.29) (Supplementary Figure S5). Five of these studies were non-NPC [6, 8–11] with 140 weekly patients and 188 three weekly patients, and there was no significant difference in the grade≥3 dermatitis between the two groups of non-NPC patients (RR=1.31, 95%CI 0.88-1.95, p=0.18). No heterogeneity was observed for dermatitis analysis.

#### Grade≥3 mucositis

**Figure 4:**
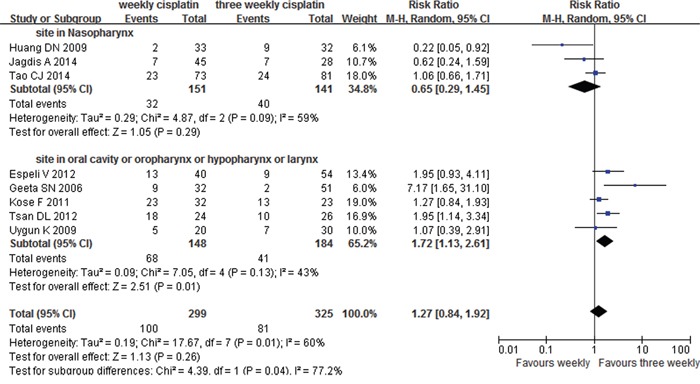
Weekly versus three weekly cisplatin chemoradiotherapy with toxicity grade ≥3 mucositis CI=confidence interval, I^2^=index of heterogeneity.

Eight articles [6, 8–10, 12–15] of 624 patients reported the data of mucosal toxicity. No obvious difference was observed for the risk of grade≥3 mucositis between the two groups. Further analysis was performed based on the disease sites. Five studies of 332 non-NPC patients (148 weekly patients and 184 three weekly patients) and three studies of 292 NPC patients (151 weekly patients and 141 three weekly patients) were included. Subgroup analyses were much interesting that patients in weekly group suffered grade ≥ 3 mucositis more easily when the primary disease located in non-nasopharynx (RR=1.72, 95%CI 1.13-2.61, p=0.01) (Figure 4) with a not significant heterogeneity of 43% (p=0.13). However, when the disease site arose in nasopharynx, patients of the two groups had similar risk (RR=0.65, 95%CI 0.29-1.45, p=0.29).

### Treatment delays or interruption

**Figure 5:**
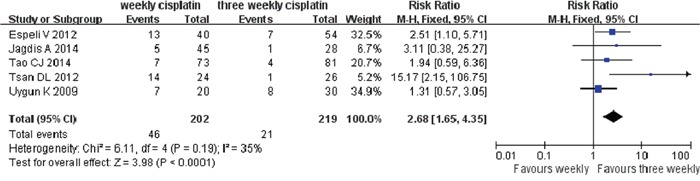
Weekly versus three weekly cisplatin chemoradiotherapy in delays or interruption of treatment CI=confidence interval, I^2^=index of heterogeneity.

Many patients suffered serious adverse effects in the process of CRT, which often resulted in delay or interrupt of treatment. We calculated the chemotherapy completion for weekly (6-8cycles) and three weekly (3cycles) from six studies [6, 9–11, 13, 15] while the forest plot revealed a noticeable heterogeneity (I^2^= 59%, p=0.03). After looking all eligible studies through, in Ho KF's study [11], there were almost 42% patients in weekly group and 30% in three weekly group received cisplatin and 5-fluorouracil (PF) induction chemotherapy prior to concurrent CRT and the majority (83%) received 2 cycles of neoadjuvant chemotherapy, which might be the source of heterogeneity. Therefore, Ho KF's study was removed from the forest analysis. Finally, five studies were enrolled with an acceptable heterogeneity (I^2^= 35%, p=0.19). It is important to note that patients in weekly group suffered more chemotherapy delay/interrupt than three weekly patients (RR=2.68, 95%CI 1.65-4.35, p<0.0001) (Figure 5).

## DISCUSSION

Currently, there has been a significant increase in the global incidence of HNC and over half of patients were diagnosed with advanced disease at the initially visit [16]. Wee J et al had reported that the addition of three weekly CDDP CRT regimen manifested 2-and 3-year overall survival rates of 85% and 80%, which was similar in the results of the Intergroup 00-99Trial with 2-year OS 82% and 3-year OS 78% [17–18]. Simultaneously, another randomized trial of 350 patients by Chan et al using weekly CDDP at 40 mg/m^2^ during the RT showed a better 5-year OS rate when compared to RT only (CRT 70.3% versus RT 58.6%, p=0.049) at the end of follow up [19]. In our analysis, a trend of better 5-year OS was found in three weekly group when compared with weekly scheme, although the two groups had similar short-term survivals (2- and 3-OS). It is reported that patients with better local control possibly possessed better survivals [17]. Therefore, we hypothesized that three weekly cisplatin CRT might improve long-term OS due to a higher rate of locoregional control though the long-term data of LRFS were not available. On the other hand, data showed that a cumulative dose of 200 mg/m^2^ might lead to similar tumor control [6, 8], thereby, it was important to achieve such dose. But the courses were always interrupted or postponed in many patients as a result of adverse effects, especially for mucosal toxicity [20]. Similarly, our meta-analysis showed that patients in weekly cisplatin group had significant difficulty in achieving targeted dose of 200 mg/m^2^ (RR=0.82, p=0.009) (Supplementary Figure S6), eventually, resulting in a worse survival benefit due to more treatment delay/interrupt and inadequate dose of cisplatin [21–22]. There is an urgent need that a longer follow-up work and higher quality of clinical trials were performed to test the ideas.

Many studies have confirmed that cisplatin acted as a radiosensitizer and cisplatin-based concurrent CRT had more toxicity than radiotherapy alone on severe (grade 3 or higher) adverse effects, whether weekly (p<0.001) or three weekly cisplatin (p=0.001) was administrated [23–26]. It was reported that up to 87% of patients treated with weekly cisplatin reached grade≥3 toxicity [18, 27] and 57% in three weekly group [14, 17, 24, 28].

One of the most serious adverse reactions is mucositis, which might be attributed to wide RT portal (extending from the skull base to the root of the neck) and significant doses of RT delivered to the mucosal surfaces due to bulky primary or cervical nodes [24]. In our study, no obvious difference was observed between the two treatments, however, subgroup analysis showed that patients with non-NPC occurred more grade≥3 mucositis when treated by weekly CRT. The reasons might be as follows: 1) More frequent administration of cisplatin. It had been reported that radiosensitization effect can be improved when with more frequent administration of cisplatin, thus, resulting in more severe mucositis [29]. Consensus opinion was supported by Tsan DL's trial. 2) The choice of hydration might account for some difference in mucosal toxicity. In a phase III randomized study, patients in three weekly group suffered significantly lower grade≥3 mucositis than weekly patients due to hydration before and after cisplatin infusion (three weekly ciaplatin vs. weekly cisplatin = 38.5% vs. 75%, p=0.012) [6], which needed more trial to confirm further.

In terms of gastrointestinal effects, patients in three weekly cisplatin group tended to occur more nausea and/or vomiting than those in weekly group (RR=0.59, p=0.06). While a phase II trial fractionated CDDP into four daily doses during the concurrent CRT to reduce the emesis rate and improved tolerability [17]. Same viewpoint was reached by other studies that spread out doses of cisplatin over several days might reduce the chemotherapy-induced gastrointestinal toxicity while still providing a beneficial antitumor effect as well [2, 6]. Therefore, clinical trials that comparing weekly cisplatin with three weekly delivered moderately are urgently needed to reach a decision scientifically.

Kidney damage was also common adverse reaction during CRT [30]. Study reported that up to 53.7% of patients treated with three weekly cisplatin suffered acute renal failure compared 35% weekly patients despite no statistical significance (p=0.07) [9]. Kose F et al had mentioned similar renal toxicity between the two different cisplatin regimens, even total cisplatin doses in three weekly group were higher than weekly group (210 mg/m^2^ vs. 162 mg/m^2^, p<0.0001). Similar conclusion was supported by others [11–13]. One of eligible studies referred to liver toxicity [12], and only grade1 toxicity was observed with no significance between weekly (0.3%) and three weekly cisplatin patients (0.3%). However, we were unable to discuss these topics due to the inconsistent assessment methods and data deficiencies from enrolled studies.

Chemotherapy administration costs and drug acquisition costs were the most significant components of treatment for inpatients and outpatients [31]. Peter G had calculated the costs of administering weekly cisplatin-based regimen for per patient with cervical cancer (inpatient setting were $8839 compared with $3590 in the outpatient setting). And the total cost of three weekly cisplatin-based CRT was $3303 for HNC patients [32]. Although no difference was reported for the mean radiotherapy overall treatment time [6, 11, 13, 15], the frequency of visiting doctors for weekly group participants tended to increase relatively. Hence, patients receiving three weekly cisplatin might be more comfortable during treatment with less administration costs.

Nevertheless, we had to mention several limitations in this study. Firstly, there were only two RCTs eligible, while the other eights were retrospective. Secondly, not all articles reported the data of OS, progression-free survival (PFS), LRFS, disease-free survival (DFS), especially the long-term survival data. Thirdly, we only analyzed the acute toxicities, whereas, chronic toxicities were not obtainable, such as xerostomia, dysphagia, hearing loss, radiation-induced brain injury and so on.

Taking together, weekly cisplatin CRT had no difference in overall survival but less comfort in treatment process compared with three weekly cisplatin regimen. It is important to proceed with a long-term follow-up of the chronic toxicities and survival events to explore which treatment could make patients benefit most. Kunieda F et al had launched a randomized phase II/III study in Japan [33], and we are looking forward to make further efforts for the research.

## MATERIALS AND METHODS

### Search methods for identification of studies

We performed a literature search in the PubMed, MEDLINE, ScineceDirect, Cochrane Library and CNKI databases published between 1982-2014. The key words were: “head and neck neoplasms” OR “head and neck cancer” OR “head and neck tumor” OR “head-neck tumors”, “cisplatin” OR “Cis-platinum” OR “Platinol” OR “CDDP”, “triweekly” OR “three weeks” OR “every three weeks”, “per week” OR “every week” OR “weekly” OR “once a week” and “randomized controlled trial” OR “randomized control trials” OR “randomized clinical trial” OR “RCT” OR “randomly allocation” OR “randomly” OR “random” OR “controlled” OR “trial” OR. We also searched for “lip” “oral cavity” “oropharyngeal” “hypopharyngeal” “nasopharyngeal” “laryngeal” “sinus” “salivary gland”, respectively. All of the eligible articles and their references were retrieved. We excluded reviews, case reports and animal experiments, moreover, meeting abstracts that had not been published full-text were also excluded. We would consider the studies as a single one if they had been published twice or more by the seam team and based on the same patient source.

### Data collection and analysis

Following information were extracted from the included studies: first author, publication year, study design, treatment protocol, number of patients, staging information, acute adverse reactions and survival events. Two authors conducted the eligibility assessment and data verification independently. If agreement could not be reached between the two authors, a third author would participate in the discussion until reaching final agreement.

Endpoints were determined as overall survival, locoregional recurrence-free survival and adverse events. RevMan 5.2 software (Cochrane Collaboration's Information Management System) was used to perform this meta-analysis. Risk ratios of adverse effect were calculated with the correspondent 95% confidence interval (CI), in addition, time-to-event data from individual trials were summarized by the log hazard ratio (HR) and its variance. If the trials did not report survival information directly, Kaplan-Meier curves were read by the Engauge Digitizer version 4.1 (free software downloaded from http://sourceforge.net) and DerSimonian-Laird random effect analysis was used to estimate the difference. Subgroup analyses were conducted according to the range of follow-up times and lesion sites. Heterogeneity was assessed by forest plots, chi-squared (χ^2^) tests and I^2^ statistic percentages. P values below 0.05 were defined as significant outcomes. Fixed-effect model was applied when homogeneity was fine (p≥0.10, I^2^≤50%), otherwise, a random-effect model was used.

## SUPPLEMENTARY FIGURES


