# Supplementary material for: A meta-analysis of weekly cisplatin versus three weekly cisplatin chemotherapy plus concurrent radiotherapy (CRT) for advanced head and neck cancer (HNC)
Source: Oncotarget. 2016 Sep 2;7(43):70185–93. doi: 10.18632/oncotarget.11824 (PMC5342545; doi:10.18632/oncotarget.11824)
Supplement: Supplementary file 1 [file oncotarget-07-70185-s001.pdf]

A meta-analysis of weekly cisplatin versus three weekly cisplatin chemotherapy plus concurrent radiotherapy (CRT) for advanced head and neck cancer (HNC)

SUPPLEMENTARY FIGURES

	weekly cisplatin		three weekly cisplatin			Risk Ratio
Study or Subgroup	Events	Total	Events	Total	Weight	M-H, Fixed, 95% CI
Geeta SN 2006	5	32	4	51	13.2%	1.99 [0.58, 6.87]
Ho KF,2008	5	24	6	27	24.2%	0.94 [0.33, 2.68]
Huang DN 2009	1	33	8	32	34.8%	0.12 [0.02, 0.91]
Jagdis A,2014	3	45	1	28	5.3%	1.87 [0.20, 17.07]
Tsan DL,2012	1	24	0	26	2.1%	3.24 [0.14, 75.91]
Uygun K,2009	3	20	6	30	20.5%	0.75 [0.21, 2.66]
**Total (95% CI)**		**178**		**194**	**100.0%**	**0.85 [0.49, 1.48]**
Total events	18		25			

Heterogeneity: Chi² = 6.63, df = 5 (P = 0.25); I² = 25%

Test for overall effect: Z = 0.57 (P = 0.57)

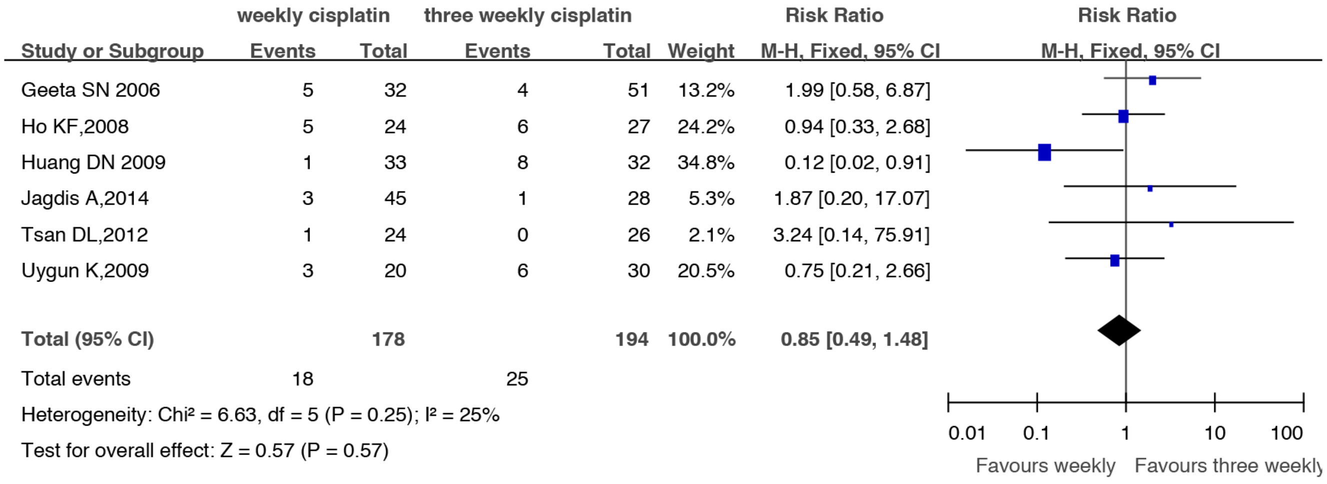

Supplementary Figure S1: Weekly versus three weekly cisplatin CRT with toxicity grade ≥3 neutropenia.

	weekly cisplatin		three weekly cisplatin			Risk Ratio	Risk Ratio
Study or Subgroup	Events	Total	Events	Total	Weight	M-H, Fixed, 95% CI	M-H, Fixed, 95% CI
Jagdis A 2014	2	45	0	28	8.2%	3.15 [0.16, 63.35]	
Tao CJ 2014	5	73	3	81	38.1%	1.85 [0.46, 7.47]	
Tsan DL 2012	0	24	0	26		Not estimable	
Uygun K 2009	1	20	5	30	53.6%	0.30 [0.04, 2.38]	
**Total (95% CI)**		**162**		**165**	**100.0%**	**1.13 [0.42, 3.01]**	
Total events	8		8				

Heterogeneity: Chi² = 2.50, df = 2 (P = 0.29); I² = 20%

Test for overall effect: Z = 0.24 (P = 0.81)

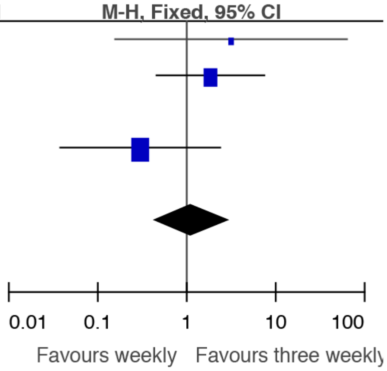

Supplementary Figure S2: Weekly versus three weekly cisplatin CRT with toxicity grade ≥3 thrombocytopenia.

	weekly cisplatin		three weekly cisplatin			Risk Ratio
Study or Subgroup	Events	Total	Events	Total	Weight	M-H, Fixed, 95% CI
Geeta SN 2006	4	32	2	51	51.8%	3.19 [0.62, 16.41]
Tao CJ 2014	2	73	0	81	15.9%	5.54 [0.27, 113.54]
Tsan DL 2012	1	24	1	26	32.3%	1.08 [0.07, 16.38]
**Total (95% CI)**		**129**		**158**	**100.0%**	**2.88 [0.84, 9.94]**
Total events	7		3			

Heterogeneity: Chi² = 0.69, df = 2 (P = 0.71); I² = 0%

Test for overall effect: Z = 1.68 (P = 0.09)

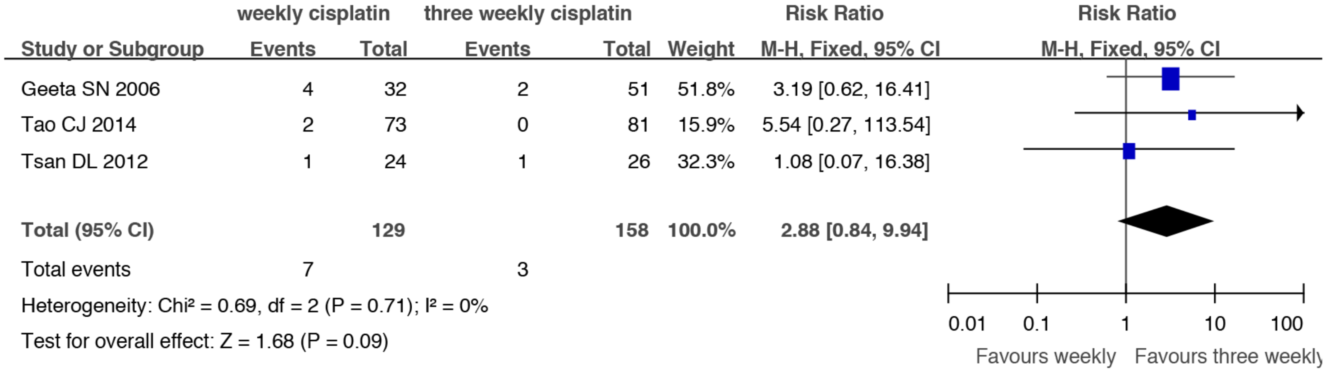

Supplementary Figure S3: Weekly versus three weekly cisplatin CRT with toxicity grade ≥3 anemia.

	weekly cisplatin		three weekly cisplatin			Risk Ratio
Study or Subgroup	Events	Total	Events	Total	Weight	M-H, Fixed, 95% CI
Ho KF,2008	2	24	6	27	18.3%	0.38 [0.08, 1.69]
Huang DN 2009	1	33	8	32	26.4%	0.12 [0.02, 0.91]
Jagdis A,2014	6	45	1	28	4.0%	3.73 [0.47, 29.40]
Tao CJ 2014	0	73	3	81	10.8%	0.16 [0.01, 3.01]
Tsan DL,2012	5	24	3	26	9.3%	1.81 [0.48, 6.76]
Uygun K,2009	4	20	12	30	31.2%	0.50 [0.19, 1.33]
**Total (95% CI)**		**219**		**224**	**100.0%**	**0.59 [0.34, 1.02]**
Total events	18		33			

Heterogeneity: Chi² = 9.41, df = 5 (P = 0.09); I² = 47%

Test for overall effect: Z = 1.87 (P = 0.06)

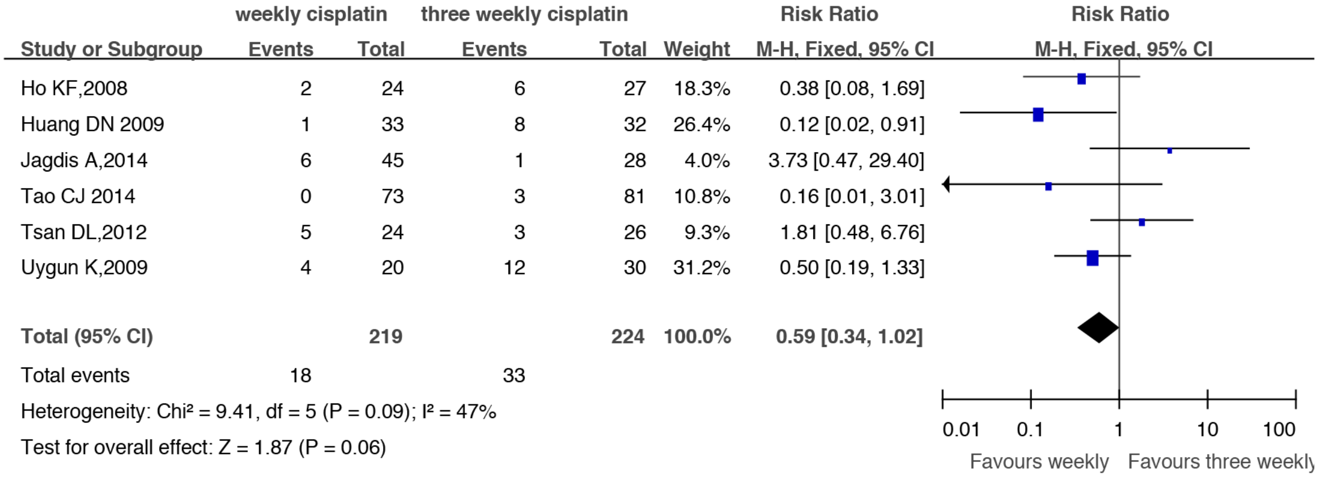

Supplementary Figure S4: Weekly versus three weekly cisplatin CRT with toxicity grade ≥3 nausea/vomiting.

	weekly cisplatin		three weekly cisplatin			Risk Ratio	Risk Ratio
Study or Subgroup	Events	Total	Events	Total	Weight	M-H, Fixed, 95% CI	M-H, Fixed, 95% CI
Espeli V,2012	13	40	20	54	50.5%	0.88 [0.50, 1.55]	
Geeta SN 2006	5	32	4	51	9.1%	1.99 [0.58, 6.87]	
Ho KF,2008	13	24	6	27	16.7%	2.44 [1.10, 5.40]	
Tao CJ 2014	1	73	3	81	8.4%	0.37 [0.04, 3.48]	
Tsan DL,2012	2	24	2	26	5.7%	1.08 [0.17, 7.10]	
Uygun K,2009	3	20	4	30	9.5%	1.13 [0.28, 4.50]	
**Total (95% CI)**		**213**		**269**	**100.0%**	**1.23 [0.84, 1.82]**	
Total events	37		39				

Heterogeneity: Chi² = 5.92, df = 5 (P = 0.31); I² = 16%

Test for overall effect: Z = 1.06 (P = 0.29)

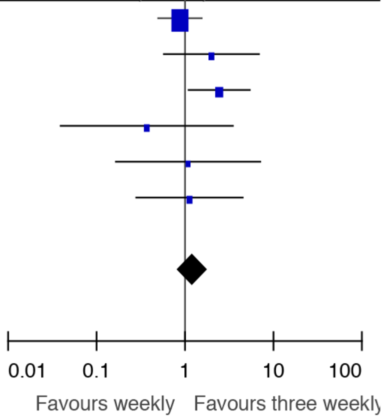

Supplementary Figure S5: Weekly versus three weekly cisplatin CRT with toxicity grade ≥3 dermatitis.

	weekly cisplatin		three weekly cisplatin			Risk Ratio
Study or Subgroup	Events	Total	Events	Total	Weight	M-H, Fixed, 95% CI
Espeli V 2012	27	40	47	54	37.5%	0.78 [0.61, 0.98]
Ho KF 2008	13	24	16	27	14.1%	0.91 [0.56, 1.48]
Jagdis A 2014	36	45	24	28	27.7%	0.93 [0.76, 1.15]
Tsan DL 2012	15	24	23	26	20.7%	0.71 [0.50, 0.99]
**Total (95% CI)**		**133**		**135**	**100.0%**	**0.82 [0.71, 0.95]**
Total events	91		110			

Heterogeneity: $Chi^2 = 2.56$, $df = 3$ ($P = 0.46$); $I^2 = 0\%$

Test for overall effect: $Z = 2.63$ ($P = 0.009$)

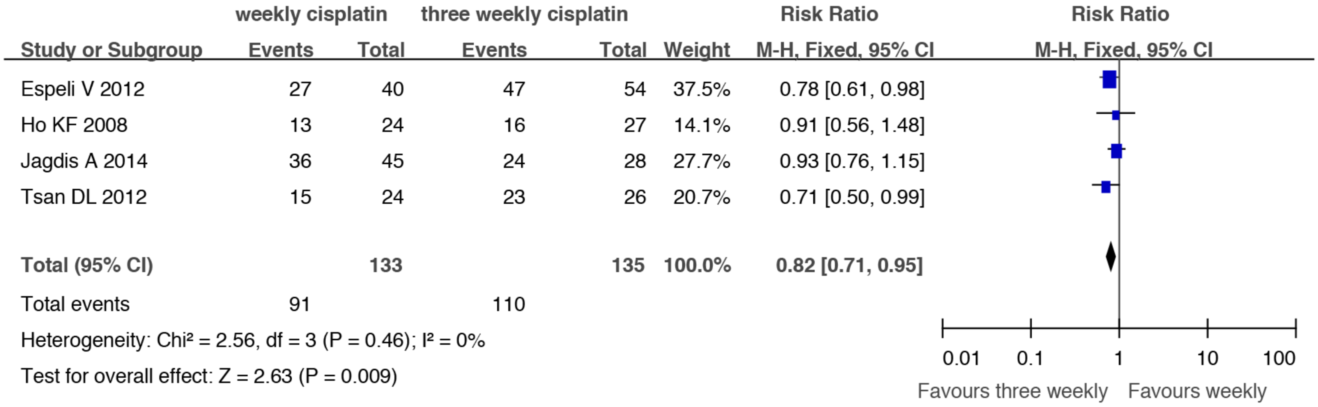

Supplementary Figure S6: Weekly versus three weekly cisplatin CRT in receiving cisplatin dose ≥ 200mg/m².
